# Dropout from a maternal and newborn continuum of care after antenatal care booking and its associated factors in Debre Berhan town, northeast Ethiopia

**DOI:** 10.3389/fmed.2022.950901

**Published:** 2022-09-29

**Authors:** Mesfin Tadese, Saba Desta Tessema, Dawit Aklilu, Getu Engida Wake, Getaneh Baye Mulu

**Affiliations:** ^1^Department of Midwifery, School of Nursing and Midwifery, Asrat Woldeyes Health Science Campus, Debre Berhan University, Debre Berhan, Ethiopia; ^2^Department of Nursing, School of Nursing and Midwifery, Asrat Woldeyes Health Science Campus, Debre Berhan University, Debre Berhan, Ethiopia

**Keywords:** dropout, continuum of care, maternal, newborn, factors

## Abstract

**Background:**

Continuum of care (CoC) is the continuity of care from the beginning of pregnancy to the postnatal period to improve maternal, neonatal, and child health. Dropout from the maternal CoC remains a public health challenge in Ethiopia. There are limited studies on women who dropped out of the CoC. The available studies have focused on the time dimension of the CoC, and there is a paucity of data on the place dimension of the CoC. Thus, this study aimed to determine the prevalence of dropout from the maternal CoC and its associated factors in Debre Berhan town, northeast Ethiopia.

**Methods:**

A community-based cross-sectional study design was conducted among 842 mothers from September to October 2020. A cluster sampling technique was applied, and data were collected through face-to-face interviews using a structured and pre-tested questionnaire. Data were cleaned and entered into EpiData version 4.6 and exported to SPSS version 25 for analysis. Descriptive statistics, and bivariable and multivariable logistic regression analyses were performed to summarize the findings, and a *p*-value of <0.05 was considered statistically significant.

**Result:**

The overall prevalence of dropout from the maternity continuum of care was 69.1% [95% CI (66.0–72.3)]. The prevalence of dropout from ANC, skilled birth attendant, and PNC visits was 45.4, 0.5, and 48.7%, respectively. Rural residents, partners' level of education, monthly income, the timing of the first ANC visit, antenatal counseling about a continuum of care, and the level of satisfaction with the service delivery were significantly associated with ANC dropout. Maternal age and occupation, partners' age, media exposure, parity, the timing of the first ANC visit, the place of ANC visit, and the time spent for an ANC visit were significantly associated with dropout from PNC visits. Husbands' occupation, monthly income, number of alive children, the timing of the first ANC visit, and the time spent for an ANC visit had a statistically significant association with dropout from the maternity CoC.

**Conclusion:**

Dropout from the CoC in the study area was high. Socioeconomic development, partner involvement, antenatal counseling, efficient service delivery, and media exposure are vital to improving the high dropout rate from the maternal continuum of care.

## Introduction

Continuum of care is defined as the continuity of care from pregnancy to childbirth and the postpartum period, that is, the use of antenatal care (ANC) during pregnancy, a skilled birth attendant at delivery, and postnatal care (PNC) after delivery. The concept suggests that maternal health and newborn health are closely related and hence should be managed in an integrated way. This model has two dimensions: a time dimension (continuity of care over time) and a place dimension (integrated service delivery provided by health facilities and communities). The maternity continuum of care is one of the key program strategies to minimize maternal and newborn deaths and to improve maternal and neonatal health outcomes and wellbeing (1).

Globally, in 2020, the maternal mortality ratio was 152 deaths per 100,000 live births, slightly higher than 151 deaths in 2019. This trajectory further projects 133 deaths in 2030, nearly double the SDG target 3.1 (2). In addition, in 2019, 2.4 million children died in their first month of life. Approximately 6,700 neonatal deaths occur every day. A third of deaths occur within the first 24 h of birth, and three-quarters (75%) occur in the first week of life (3). According to the Ethiopian Demographic Health Survey (EDHS), the pregnancy-related maternal mortality ratio was 412 deaths per 100,000 live births, and the neonatal mortality rate was 29 deaths per 1,000 live births (4). Most of these deaths were preventable with access to high-quality care provided by competent skilled health professionals during pregnancy (ANC), childbirth (intrapartum care), and the postpartum period (PNC) (5).

The prevalence of dropout from a continuum of care varies globally, with a higher prevalence rate in developing countries. About 38.1% of women in Nigeria dropped out of skilled delivery, and 50.8% dropped out of the PNC (6). In Tanzania, only 10.3% of women completed the recommended contacts through the entire continuum of care (7). In Cambodia, over 90% of women had at least one ANC visit, 60% had four or more visits, 74% had skilled birth attendance, and 71% had at least one PNC visit (1). The prevalence of dropout from a continuum of maternal care in northwest Ethiopia was 55% (8). A secondary analysis of the EDHS found that 44.6%, 46.5%, and 87.1% of mothers dropped out of ANC, institutional delivery, and PNC visit before completing the recommended number of visits, respectively (9). Furthermore, in Ethiopia, only 37.2% (10) and 21.6% (11) of women had a full range of continuum of care in Debre Berhan town and North Gondar zone, respectively.

Shortage of medical equipment, drugs, and other supplies; lack and cost of transport; culture; and previous maternal experiences, as well as maternal sociodemographic factors, that is, age at the time of pregnancy, rural residence, income, and low level of education, was a barrier to seeking and completing antenatal care services (12). Long distance to a health facility, poor antenatal counseling, lack of autonomy in healthcare decisions, no media exposure, inadequate provision of health education and promotion, poor knowledge of pregnancy complications, and danger signs were also among the factors influencing the higher rate of dropout from the continuum of care (6–8, 11).

Completing the maternal and neonatal CoC helps achieve the Sustainable Development Goal (SDG) 3 through reducing severe maternal and neonatal morbidity rates, mortality rates, and long-term physical and psychological complications. ANC attendance promotes early detection and treatment of complications, that is, hypertension in pregnancy, anemia, intrauterine growth retardation, syphilis, HIV, and mental health problems, which result in proper management during delivery and puerperium (13). A combination of universal coverage of all the packages (99%) of care could avert an estimated 41–72% of neonatal deaths worldwide. In addition, a coverage at 90% averts 18–37% of neonatal deaths (14). However, in Ethiopia, few women complete the recommended continuum of care. In addition, there is limited evidence supporting the dropout of women from the CoC. Although previous studies in Ethiopia have focused on the time dimension of the CoC, there is a paucity of data regarding the place dimension of the continuum of care. The primary objective of this study is to assess the extent of dropout within and across stages of the continuum of care from pregnancy to delivery and the postnatal period. The secondary objective is to identify independent predictors of the dropout when moving from one stage of the CoC to the next.

## Methods and materials

### Study area, period, and design

This is a community-based cross-sectional study design conducted in Debre Berhan town from September to October 2020. Debre Berhan is the capital city of the North Shewa zone of the Amhara Region and is about 130 kilometers northeast of Addis Ababa. The size of this town is the highest in Africa, with an elevation of 2,840 meters. According to the 2019 report, the town contained nine kebeles (the smallest administrative unit) with a total population of 114,652, of whom 62,809 were female. About 39,066 female individuals were within the age range of 15–49 years. Debre Berhan has two hospitals, three health centers, and 14 clinics, all providing maternal and child health services.

### Sample size and sampling procedure

The required sample size was computed using OpenEpi Software for Epidemiologic Statistics version 3.03 with the assumptions of 95% confidence level, 5% degree of precision, 38.1% dropout from skilled delivery (6), design effect 2, and 10% compensation for possible non-response. The final sample size was estimated at 798.

Study participants were selected by the cluster sampling technique. Of the nine kebeles, five were chosen by using a lottery method, and all mothers in each cluster who fulfill the inclusion criteria were included in the study. A total of 867 eligible women were found in the selected clusters using the data from health extension workers (registration book), and all were included in the study.

### Inclusion and exclusion criteria

Women who gave birth at least one time in the last 12 months preceding the survey, who had booked for ANC, and who were at or beyond 6 weeks after birth were included. Women who had not lived permanently in the study area for at least 6 months at the time of data collection were excluded from the study.

### Study variables

Dropout from the maternity continuum of care is the outcome variable. The exposure variables include sociodemographic factors (age, level of education, income, and media exposure), reproductive and obstetrics factors (parity, number of alive children, history of abortion, and plan for the current pregnancy), and other health service-related characteristics (awareness about CoC, level of distance of health facility, and satisfaction with service delivery).

### Definition of terms

Antenatal care (ANC) dropout: This dropout is considered if a woman had less than four ANC visits during her most recent pregnancy.

Skilled birth attendant (SBA) dropout: It is considered if a women had four or more ANC visits but did not seek an SBA (delivery was not assisted by healthcare professionals, i.e., midwives, nurses, doctors, and/or health officers).

Postnatal care (PNC) dropout: It is considered if a woman sought an SBA but did not attend PNC visits within the first 6 weeks of delivery.

Dropout from maternity continuum of care: It is considered if a woman drops out of ANC, skilled birth attendant, and/or postnatal care visits.

### Data collection tool and procedure

The data were collected through face-to-face interviews using a structured and pre-tested questionnaire at the participant's home. The questionnaire was prepared based on the Ethiopian Demographic Health Survey (EDHS) questionnaire and measurement tools used in previous studies (4, 6, 7, 9). A total of 33 open- and closed-ended questions were included in the questionnaire. The interview took an average of 15–20 min.

### Data quality assurance

A total of six data collectors (four midwives and two nurses) and two supervisions were involved in the data collection. The data collectors and supervisors received a 1-day orientation on the objective, approach, data collection tool, and procedure by the principal investigators. Back and forth questionnaire translation (from English to Amharic, and vice versa) was performed to check the consistency of the questions with the original meaning. Before the actual data collection, a pre-test was conducted on 5% of the participants (43 women), and the required amendments were considered following the result. The supervisors and principal investigators closely observed the completeness, coherence, and clarity of the data across the entire data collection period.

### Data processing and analysis

The data were cleaned, recoded, and entered into EpiData version 4.6 and transferred to IBM SPSS version 25.0 statistical software for analysis. The principal investigator randomly picked one questionnaire and compared it with the corresponding entered data for quality control. Descriptive findings were presented using narratives, figures, and tables. Initially, bivariable logistic regression analyses were carried out for all the variables listed in the descriptive table to select candidates for multivariable logistic regression analysis based on a *p*-value of <0.25. A multivariable logistic regression model was fitted for three outcome variables (dropout from ANC, PNC, and the overall continuum of care). Hosmer–Lemeshow goodness of fit was tested for model fitness. Multicollinearity between explanatory variables was also checked. A two-sided confidence level was set at a 95% confidence interval, and statistical significance was declared at a *p*-value of <0.05.

### Ethical consideration

The study was approved by the Institutional Review Board of the School of Medicine and Health Sciences, the University of Gondar. A formal permission was obtained from the Debre Berhan town health office. Participation was voluntary, and an informed written consent was obtained from the study participants. Those who cannot read and write were asked to thumbprint the consent form after the information was read. The names of the participants were not recorded, and confidentiality was secured. The study was conducted following the ethical principles of the Declaration of Helsinki.

## Result

### Demographic characteristics of respondents

A total of 842 women were included in this study with a response rate of 97.1%. The mean age ± standard deviation (SD) of women who completed maternity care and the dropout continuum of care was 29.58 ± 3.96 and 30.65 ± 5.43 years, respectively. About 80.8% of women who dropped out of the continuum of care were older than 35 years. More than two-thirds (69.8%) of Christians and 87% of rural residents dropped out of the maternal continuum of care. One-third (32.7%) of married women have completed maternity care, while 82.8% of single women did not. In addition, 42.5% of mothers in the middle-income tertile have completed the continuum of care, whereas 84.6% of mothers in the low-income tertile dropped out of maternity care (Table 1).

**Table 1:** Distribution of sociodemographic data by dropout from continuum of care in Debre Berhan town, northeast Ethiopia.

**Variables**	**Category**	**Dropout continuum of care**, ***n*** **(%)**		***P*-value**
		**No (260)**	**Yes (582)**	
Age	≤ 20 years	4 (20.0)	16(80.0)	0.000*
	21–34 years	218 (34.9)	406 (65.1)	
	≥35 years	38 (19.2)	160 (80.8)	
Residence	Urban	242 (34.4)	462 (65.6)	0.001
	Rural	18 (13.0)	120 (87.0)	
Religion	Christian	220 (30.2)	508 (69.8)	0.296
	Muslim	40 (35.1)	74 (64.9)	
Ethnicity	Amhara	208 (31.9)	444 (68.1)	0.234
	Others©	52 (27.4)	138 (72.6)	
Mother's education	No formal education	60 (22.2)	210 (77.8)	0.000
	Primary	12 (16.2)	62 (83.8)	
	Secondary	48 (28.6)	120 (71.4)	
	Higher education	140 (42.4)	190 (57.6)	
Mother's occupation	Housewife	62 (24.6)	190 (75.4)	0.000
	Gov't employee	94 (47.0)	106 (53.0)	
	Self-employed	104 (26.7)	286 (73.3)	
Marital status	Married	246 (32.7)	506 (67.3)	0.004*
	Single	10 (17.2)	48 (82.8)	
	Divorced and widowed	4 (12.5)	28 (87.5)	
Age of partner (*n* = 784)	20–29 years	24 (40.0)	36 (60.0)	0.000*
	30–39 years	160 (35.9)	286 (64.1)	
	40–49 years	66 (25.8)	190 (74.2)	
	≥50 years	0 (0)	22 (100.0)	
Education of partner (*n* = 784)	No formal education	32 (19.0)	136 (81.0)	0.000
	Primary	10 (14.7)	58 (85.3)	
	Secondary	42 (28.0)	108 (72.0)	
	Higher education	166 (41.7)	232 (58.3)	
Occupation of partner (*n* = 784)	Farmer	10 (9.1)	100 (90.9)	0.000
	Gov't employee	126 (42.9)	168 (57.1)	
	Self-employed	114 (30.0)	266 (70.0)	
Family monthly income (ETB)	Lower tertile	50 (15.4)	274 (84.6)	0.000
	Middle tertile	102 (42.5)	138 (57.5)	
	Higher tertile	108 (38.8)	170 (61.2)	
Media exposure	Yes	258 (31.7)	556 (68.3)	0.006*
	No	2 (7.1)	26 (92.9)	
Types of media (*n* = 814)	Mass-media (TV/Radio)	200 (28.7)	496 (71.3)	0.000
	Social-media	58 (49.2)	60 (50.8)	

Others©, Oromo, Tigray, Gurage, and Argobba; ETB, Ethiopian birr. *Fisher's exact test.

### Reproductive and obstetric characteristics

Nearly two-thirds (31.8%) of low multiparous (2–4) women completed maternity care, and all (100%) grand multiparous women dropped out of the continuum of care. Most (86%) of the mothers with unplanned and unwanted pregnancies dropped out of the maternal continuum of care. Furthermore, 70.9% of women with a history of abortion dropped out of maternity care (Table 2).

**Table 2:** Reproductive and obstetric characteristics by dropout from continuum of care in Debre Berhan town, northeast Ethiopia.

**Variables**	**Category**	**Dropout continuum of care**, ***n*** **(%)**		***P*-value**
		**No (260)**	**Yes (582)**	
Gravidity	1	84 (32.8)	172 (67.2)	0.001*
	2–4	172 (32.1)	364 (67.9)	
	≥ 5	4 (8.0)	26 (52.0)	
Parity	Primipara	92 (33.1)	186 (66.9)	0.001*
	Low multipara (2–4)	168 (31.8)	366 (68.5)	
	Grand multipara (≥5)	0 (0.0)	30 (100.0)	
Pregnancy planned	Yes	244 (33.9)	476 (66.1)	0.000
	No	16 (13.1)	106 (86.9)	
Pregnancy wanted	Yes	246 (33.3)	492 (66.7)	0.000
	No	14 (13.5)	90 (86.5)	
Partner support	Yes	238 (33.4)	474 (66.7)	0.000
	No	22 (16.9)	108 (83.1)	
Number of alive children	1	94 (33.6)	186 (66.4)	0.000
	2–3	152 (33.9)	296 (66.1)	
	≥4	14 (12.3)	100 (87.7)	
History of abortion	Yes	32 (29.1)	78 (70.9)	0.663
	No	228 (31.1)	504 (68.9)	

### Maternal healthcare service utilization

Among the study participants, 68% of women who heard about continuum of care dropped out of the care. The majority (85%) of women with late first antenatal care (ANC) follow-ups dropped out of the maternal continuum of care. Almost all (95%) women who were not counseled about a continuum of care dropped out of the maternity care. In addition, 88.5% of women who were dissatisfied with the service delivery and 91.5% who were dissatisfied with the information provided dropped out of the maternal continuum of care (Table 3).

**Table 3:** Maternal healthcare service utilization by dropout from continuum of care in Debre Berhan town, northeast Ethiopia.

**Variables**	**Category**	**Dropout continuum of care, *n* (%)**	***P*-value**
		**No (260)**	**Yes (582)**	
Ever heard about continuum of care	Yes	260 (31.9)	556 (68.1)	0.000*
	No	0 (0.0)	26 (100.0)	
Partner ever heard about a continuum of care	Yes	230 (36.6)	398 (63.4)	0.000*
	No	4 (8.3)	44 (91.7)	
	I don't know	26 (15.7)	140 (84.3)	
Antenatal care (at least 4 visit)	Yes	260 (56.5)	200 (43.5)	0.000*
	No	0 (0.0)	382 (100.0)	
Time of first ANC visit	≤ 16 weeks	196 (47.6)	216 (52.4)	0.000
	>16 weeks	64 (14.9)	366 (85.1)	
Place of ANC visit	Public health center	168 (30.7)	380 (69.3)	0.895
	Public hospital	68 (30.6)	154 (69.4)	
	Private institutions	24 (33.3)	48 (66.7)	
Counseled about continuum of care during ANC visit	Yes	256 (33.6)	506 (66.4)	0.000*
	No	4 (5.0)	76 (95.0)	
Time spent for ANC	≤ 15 min	34 (50.0)	34 (50.0)	0.000
	>15 min	226 (29.2)	548 (70.8)	
Place of birth	Home	0 (0)	4 (100)	0.180*
	Health facility	260 (31.0)	578 (69.0)	
Postnatal visit	Yes	260 (60.7)	168 (39.3)	0.000*
	No	0 (0.0)	414 (100.0)	
Distance of nearest health facility	≤ 15 min	8 (40.0)	12 (60.0)	0.006
	16–30 min	148 (35.6)	268 (64.4)	
	>30 min	104 (25.6)	302 (74.4)	
Level of satisfaction with service delivery	Satisfied	242 (35.3)	444 (64.7)	0.000
	Dissatisfied	18 (11.5)	138 (88.5)	
Level of satisfaction with information provided	Satisfied	248 (35.4)	452 (64.6)	0.000
	Dissatisfied	12 (8.5)	130 (91.5)	
Level of satisfaction with behavioral aspects	Satisfied	244 (35.3)	448 (64.7)	0.000
	Dissatisfied	16 (10.7)	134 (89.3)	

*Fisher's exact test.

### The proportion of dropouts from the continuum of maternity care

**Figure 1:**
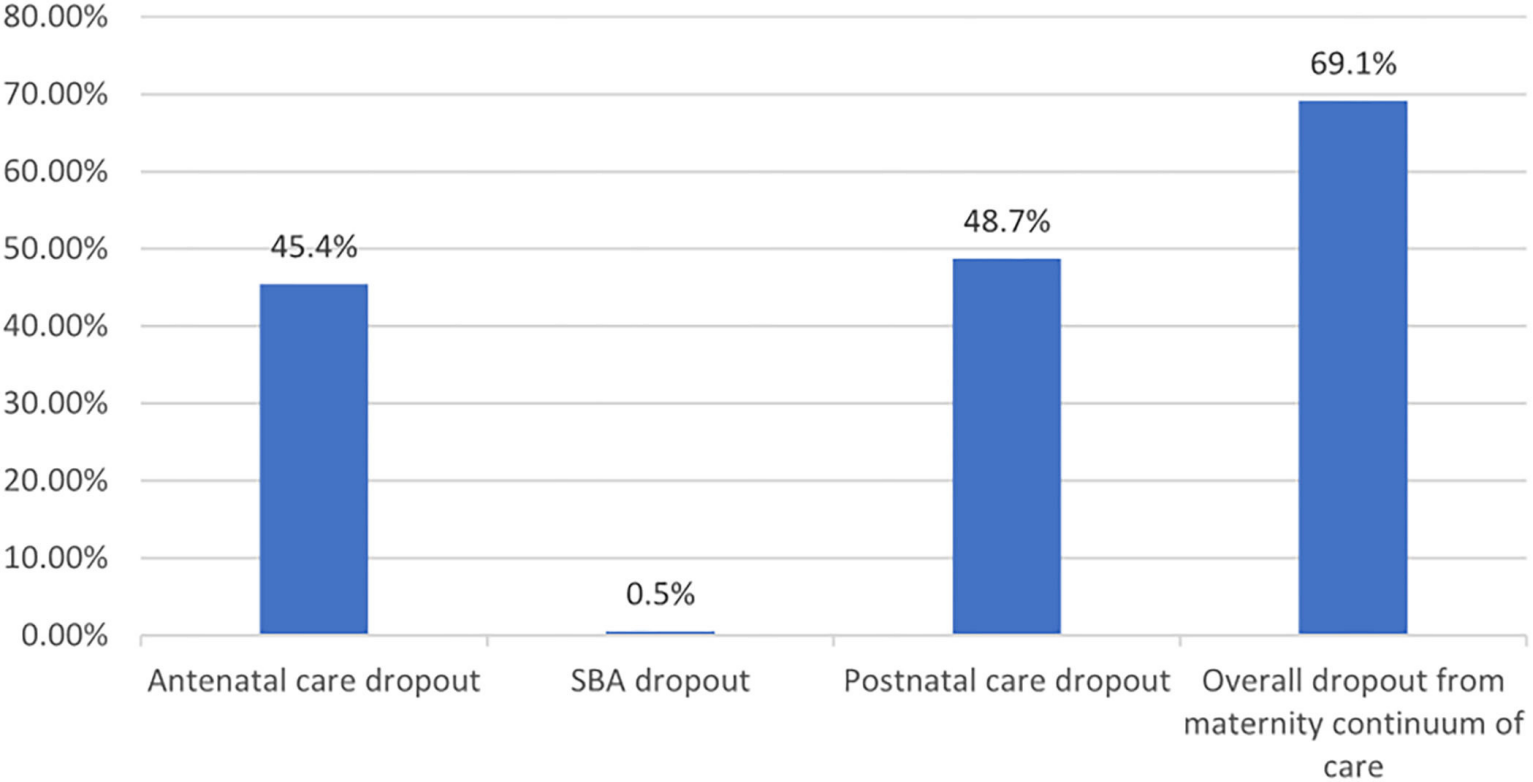
Dropout from the maternal continuum of care along the continuum care pathway in Debre Berhan Town Northeast Ethiopia.

The overall prevalence of dropout from the maternity continuum of care was 582 (69.1%) [95% CI (66.0–72.3)]. In addition, 382 (45.4%) mothers had less than four ANC visits, and 410 (48.7%) of them had no postnatal checkup after meeting with a skilled birth attendant (Figure 1).

### Factors associated with dropout from maternity continuum of care

#### Factors associated with antenatal care (ANC) dropout

Bivariable logistic regression analysis were performed to identify a candidate variable for multivariable analysis at a *p*-value <0.25. Rural residents, partners' level of education, monthly income, the timing of the first ANC visit, antenatal counseling about a continuum of care, and the level of satisfaction with the service delivery were significantly associated with antenatal care dropout (Table 4).

**Table 4:** Bivariable and multivariable logistic regression analyses of variables with antenatal care dropout in northeast Ethiopia.

**Variables**	**Category**	**COR (95% CI)**	**AOR (95% CI)**	***P*-value**
Age	≤ 20 years	1.79 (0.66–4.85)	1.53 (0.31–7.59)	
	21–34 years	0.53 (0.38–0.74)	0.75 (0.42–1.33)	0.322
	≥35 years	1	1	
Residence	Urban	1	1	
	Rural	3.08 (2.09–4.55)	3.93 (1.14–13.5)	0.030*
Mother's education	No formal education	3.06 (2.18–4.28)	0.66 (0.33–1.31)	0.230
	Primary	4.65 (2.72–7.99)	0.99 (0.41–2.44)	
	Secondary	1.76 (1.19–2.58)	0.73 (0.38–1.44)	
	Higher education	1	1	
Mother's occupation	Housewife	1	1	
	Gov't employee	0.37 (0.25–0.55)	1.30 (0.63–2.70)	
	Self-employed	1.07 (0.78–1.47)	1.37 (0.84–2.24)	0.210
Age of partner	20–29 years	1	1	
	30–39 years	0.87 (0.50–1.51)	1.19 (0.55–2.60)	
	40–49 years	1.75 (0.99–3.10)	1.64 (0.65–4.14)	
	≥50 years	1.80 (0.67–4.82)	4.14 (0.84–20.5)	0.082
Education of partner	No formal education	4.86 (3.30–7.16)	0.99 (0.46–2.11)	
	Primary	3.08 (1.82–5.21)	0.93 (0.39–2.20)	
	Secondary	2.24 (1.53–3.300	1.98 (1.05–3.74)	0.035*
	Higher education	1	1	
Occupation of partner	Farmer	2.48 (1.58–3.90)	2.06 (0.53–8.08)	
	Gov't employee	0.43 (0.31–0.59)	0.63 (0.37–1.09)	0.102
	Self-employed	1	1	
Family monthly income (ETB)	Lower tertile	3.57 (2.55–5.01)	2.96 (1.62–5.42)	0.000*
	Middle tertile	1.34 (0.93–1.92)	1.80 (1.06–3.06)	0.029*
	Higher tertile	1	1	
Pregnancy planned	Yes	1	1	
	No	5.04 (3.22–7.87)	0.91 (0.39–2.10)	0.820
Partner support	Yes	1	1	
	No	4.21 (2.76–6.39)	1.85 (0.85–4.02)	0.122
Number of alive children	1	1	1	
	2 – 3	0.90 (0.67–1.22)	0.96 (0.59–1.54)	
	≥4	1.68 (0.08–2.61)	0.86 (0.40–1.82)	0.691
History of abortion	Yes	1	1	
	No	0.71 (0.48–1.06)	0.92 (0.52–1.61)	0.766
Time of first ANC visit	≤ 16 weeks	1	1	
	> 16 weeks	10.9 (7.88–15.1)	13.2 (8.46–20.6)	0.000*
Place of ANC visit	Public health center	2.59 (1.48–4.53)	1.44 (0.67–3.10)	
	Public hospital	2.95 (1.63–5.34)	1.88 (0.83–4.23)	0.129
	Private institutions	1	1	
Counseling about a CoC during ANC visit	Yes	1	1	
	No	13.1 (6.23–27.6)	9.56 (2.72–33.6)	0.000*
Time spent for ANC	≤ 15 min	1	1	
	>15 min	1.38 (0.83–2.28)	0.66 (0.33–1.33)	0.242
Distance of nearest health facility	≤ 15 min	‘1	1	
	16–30 min	1.75 (0.66–4.63)	4.71 (0.99–22.3)	0.051
	>30 min	2.22 (0.84–5.89)	1.72 (0.36–8.13)	
Level of satisfaction with service delivery	Satisfied	1	1	
	Dissatisfied	4.58 (3.09–6.77)	2.85 (1.59–5.11)	0.000*

*Statistically significant at a p-value <0.05.

Mothers whose husbands attended secondary education were approximately two times more likely to drop out from the ANC than those who attended higher education [AOR (CI) = 1.98 (1.05–3.74)]. The odds of antenatal care dropout were four times higher among rural residents [AOR (CI) = 3.93 (1.14–13.5)]. Mothers in the low-income tertile were three times more likely to drop out of antenatal care than those in higher income tertiles [AOR (CI) = 2.96 (1.62–5.42)]. Women who started ANC attendance late had higher odds of antenatal care dropout [AOR (CI) = 13.2 (8.46–20.6)]. Women who were not counseled about a continuum of care during ANC visits were more likely to drop out of antenatal care than their counterparts [AOR (CI) = 9.56 (2.72–33.6)]. The odds of ANC dropout among women who were dissatisfied with the service delivery was three times higher than those who were satisfied [AOR (CI) = 2.85 (1.59–5.11)] (Table 4).

#### Factors associated with postnatal care (PNC) dropout

Maternal age, husbands' age, mothers' occupation, media exposure, parity, the timing of the first ANC, the place of ANC visit, and the time spent to attend an ANC visit were significant predictors of postnatal care dropout (Table 5).

**Table 5:** Factors associated with post-natal care dropout in northeast Ethiopia.

**Variables**	**Category**	**COR (95% CI)**	**AOR (95% CI)**	***P*-value**
Age	≤ 20 years	0.65 (0.26–1.63)	0.59 (0.15–2.37)	
	21–34 years	0.53 (0.38–0.73)	0.60 (0.38–0.94)	0.026*
	≥35 years	1	1	
Residence	Urban	1	1	
	Rural	1.26 (0.88–1.83)	0.67 (0.24–1.88)	0.453
Mother's education	No formal education	1.51 (1.09–2.09)	0.96 (0.55–1.68)	0.500
	Primary	1.56 (0.94–2.58)	0.76 (0.35–1.67)	
	Secondary	1.32 (0.91–1.92)	0.99 (0.59–1.67)	
	Higher education	1	1	
Mother's occupation	Housewife	1	1	
	Gov't employee	0.54 (0.37–0.79)	0.49 (0.27–0.88)	0.017*
	Self-employed	0.86 (0.63–1.18)	0.90 (0.60–1.36)	
Age of partner	20–29 years	1	1	
	30–39 years	2.40 (1.32–4.39)	3.71 (1.74–7.89)	0.001*
	40–49 years	3.32 (1.78–6.19)	3.07 (1.33–7.08)	0.008*
	≥50 years	3.30 (1.19–9.11)	2.13 (0.53–8.51)	
Education of partner	No formal education	1.09 (0.76–1.57)	0.35 (0.18–1.68)	0.312
	Primary	2.31 (1.35–3.95)	1.33 (0.61–2.91)	
	Secondary	1.37 (0.94–1.99)	1.07 (0.65–1.78)	
	Higher education	1	1	
Occupation of partner	Farmer	1.35 (0.88–2.06)	2.41 (0.76–7.68)	0.137
	Gov't employee	0.80 (0.59–1.09)	0.96 (0.63–1.48)	
	Self-employed	1		
Family monthly income (ETB)	Lower tertile	1.38 (1.01–1.91)	1.23 (0.75–2.01)	
	Middle tertile	0.84 (0.59–1.19)	0.75 (0.49–1.15)	0.184
	Higher tertile	1	1	
Media exposure	Yes	1	1	0.002*
	No	6.65 (2.29–19.3)	12.6 (2.63–60.2)	
Parity	Primipara	1	1	
	Low multipara	1.06 (0.79–1.42)	1.03 (0.71–1.51)	
	Grand multipara	16.4 (3.83–70.2)	14.5 (3.02–69.5)	0.001*
Pregnancy planned	Yes	0.66 (0.45–0.98)	1.78 (0.82–3.82)	0.143
	No	1	1	
Partner support	Yes	0.63 (0.43–0.92)	0.69 (0.35–1.38)	0.295
	No	1	1	
Ever heard about continuum of care	Yes	0.41 (0.17–0.96)	0.39 (0.06–2.81)	0.354
	No	1	1	
Antenatal care (4 or more visit)	Yes	0.63 (0.48–0.83)	1.16 (0.78–1.73)	0.456
	No	1	1	
Counseled about continuum of care during ANC visit	Yes	0.33 (0.19–0.54)	0.62 (0.29–1.29)	0.201
	No	1	1	
Time of first ANC visit	≤ 16 weeks	1	1	
	>16 weeks	1.79 (1.37–2.36)	1.72 (1.18–2.49)	0.004*
Place of ANC visit	Public health center	0.56 (0.34–0.94)	0.46 (0.26–0.82)	0.008*
	Public hospital	0.60 (0.35–1.04)	0.42 (0.23–0.79)	0.007*
	Private institutions	1	1	
Time spent for ANC	≤ 15 min	1	1	
	>15 min	2.10 (1.24–3.56)	1.93 (1.07–3.48)	0.028*
Level of satisfaction with service delivery	Satisfied	0.56 (0.39–0.80)	1.03 (0.57–1.85)	0.295
	Dissatisfied	1	1	
Level of satisfaction with information provided	Satisfied	0.49 (0.33–0.71)	0.57 (0.30–1.09)	0.094
	Dissatisfied	1	1	

*Statistically significant at a p-value <0.05.

Mothers aged between 21 and 34 years had a 40% less risk of having PNC dropout than those aged older than 35 years [AOR (CI) = 0.60 (0.38–0.94)]. Similarly, mothers whose husbands were aged between 40 and 49 years were three times more likely to drop out of the PNC than those aged between 20 and 29 years [AOR (CI) = 3.07 (1.33–7.08)]. Women who were government employees were less likely to have PNC dropouts than those who were housewives [AOR (CI) = 0.49 (0.27–0.88)]. Women who were not exposed to media were more likely to drop out of PNC [AOR (CI) = 12.6 (2.63–60.2)]. Grand multiparity significantly increased the odds of PNC dropout by 14-fold [AOR (CI) = 14.5 (3.02–69.5)]. Women who started the ANC follow-up after 16 weeks were more likely to drop out of the PNC than those who started earlier [AOR (CI) = 1.72 (1.18–2.49)]. Women who attended public health facilities had reduced odds of PNC dropout compared with those who attend private health facilities [AOR (CI) = 0.46 (0.26–0.82)]. Women who spent approximately more than 15 min for ANC visits were two times more likely to drop out of the PNC than those who spent <15 min [AOR (CI) = 1.93 (1.07–3.48)] (Table 5).

#### Factors associated with dropout from maternity continuum of care

According to the multivariable logistic regression analysis, husbands' occupation, family monthly income, the number of children alive, the timing of the first ANC visit, and the time spent for an ANC visit had a statistically significant association with dropout from a continuum of care (Table 6).

**Table 6:** Factors associated with dropout from maternity care continuum in northeast Ethiopia.

**Variables**	**Category**	**COR (95% CI)**	**AOR (95% CI)**	**P-value**
Residence	Urban	1	1	
	Rural	3.49 (2.08–5.87)	0.87 (0.28–2.71)	0.814
Mother's education	No formal education	2.58 (1.79–3.69)	0.75 (0.41–1.37)	0.354
	Primary	3.81 (1.97–7.33)	0.68 (0.27–1.74)	
	Secondary	1.84 (1.24–2.75)	0.86 (0.48–1.53)	
	Higher education	1	1	
Mother's occupation	Housewife	1	1	
	Gov't employee	0.37 (0.25–0.55)	0.61 (0.33–1.13)	0.118
	Self-employed	0.89 (0.62–1.29)	0.86 (0.54–1.37)	
Education of partner	No formal education	3.04 (1.97–4.69)	0.49 (0.24–1.04)	0.063
	Primary	4.15 (2.06–8.36)	1.28 (0.48–3.42)	
	Secondary	1.84 (1.22–2.77)	1.30 (0.73–2.32)	
	Higher education	1	1	
Occupation of partner	Farmer	4.28 (2.15–8.51)	7.39 (1.91–28.6)	0.004*
	Gov't employee	0.57 (0.42–0.78)	0.91 (0.57–1.45)	
	Self-employed	1	1	
Family monthly income (ETB)	Lower tertile	3.48 (2.37–5.12)	2.27 (1.33–3.87)	0.003*
	Middle tertile	0.86 (0.61–1.22)	0.82 (0.53–1.27)	
	Higher tertile	1	1	
Pregnancy planned	Yes	0.29 (0.17–0.51)	1.13 (0.17–7.58)	0.898
	No	1	1	
Pregnancy wanted	Yes	0.31 (0.17–0.56)	1.33 (0.17–10.4)	0.788
	No	1	1	
Partner support	Yes	0.41 (0.25–0.66)	0.78 (0.33–1.84)	0.574
	No	1	1	
Number of alive children	1	1	1	
	2–3	0.98 (0.72–1.35)	1.18 (0.79–1.77)	
	≥4	3.61 (1.96–6.65)	4.29 (2.09–8.83)	0.000*
Time of first ANC visit	≤ 16 weeks	1	1	
	>16 weeks	5.19 (3.74–7.21)	3.95 (2.65–5.88)	0.000*
Time spent for ANC	≤ 15 min	1	1	
	>15 min	2.43 (1.47–3.99)	1.91 (1.04–3.51)	0.036*
Distance of nearest health facility	≤ 15 min	1	1	
	16–30 min	1.21 (0.48–3.02)	1.42 (0.43–4.69)	0.562
	>30 min	1.94 (0.77–4.87)	1.19 (0.35–4.03)	
Level of satisfaction with service delivery	Satisfied	0.24 (90.14–0.40)	0.71 (0.32–1.58)	0.399
	Dissatisfied	1	1	
Level of satisfaction with information provided	Satisfied	0.17 (0.09–0.31)	0.38 (0.14–1.04)	0.060
	Dissatisfied	1	1	
Level of satisfaction with behavioral aspects	Satisfied	0.22 (0.13–0.38)	0.65 (0.28–1.51)	0.316
	Dissatisfied	1	1	

*Statistically significant at a p-value <0.05.

Women whose husbands were farmer was seven times more likely to drop out of the maternity care continuum [AOR (CI) = 7.39 (1.91–28.6)]. Mothers in the low-income tertile had increased odds of continuum care dropping out compared with those in higher income tertiles [AOR (CI) = 2.27 (1.33–3.87)]. Those who have two or three children alive were more likely to drop out of a maternal continuum of care than those who have only one child [AOR (CI) = 4.29 (2.09–8.83)]. The odds of dropout from a maternal continuum of care were four times higher among women who started the ANC follow-up later than those who started earlier [AOR (CI) = 3.95 (2.65–5.88)]. Women who spent more than 15 min for ANC visits were two times more likely to drop out of the maternity care continuum than those who spent <15 min [AOR (CI) = 1.91 (1.04–3.51)] (Table 6).

## Discussion

The current study determined the prevalence and factors associated with the dropout from the continuum of maternity care in Debre Berhan town. The overall prevalence of dropout from the maternity continuum of care was 69.1% [95% CI (66.0–72.3)]. The prevalence of dropout from ANC, skilled birth attendants (SBA), and PNC visits was 45.4, 0.5, and 48.7%, respectively. Rural residents, partners' level of education, monthly income, the timing of the first ANC visit, antenatal counseling about a continuum of care, and the level of satisfaction with the service delivery were significantly associated with ANC dropout. Maternal age and occupation, partners' age, media exposure, parity, the timing of the first ANC visit, the place of the ANC visit, and the time spent to attend an ANC visit were significantly associated with dropout from PNC visit. Husbands' occupation, monthly income, the number of children alive, the timing of the first ANC visit, and the time spent for an ANC visit had a statistically significant association with dropout from the maternity continuum of care.

### Dropout from the continuum of care (CoC)

The largest gap and contributor to high dropout from CoC was observed at PNC, followed by ANC and SBA. The prevalence of dropout from a continuum of care in the present study was consistent with the finding reported from Pakistan where only 27% of women received full ANC and PNC, and were assisted by a skilled birth attendant during their most recent births (15). A secondary analysis of the EDHS showed that 44.6% of women dropped out of recommended antenatal care visits (9). Similarly, in Nigeria, 50.8% of women did not attend postnatal visits after institutional delivery (6). This high CoC dropout rate in the country suggests a higher risk of maternal, infant, and neonatal mortality as many women and their children could miss essential interventions at various stages of the care continuum pathway.

However, the dropout rate of this study was lower than that in the study conducted in Nigeria, where 38.1% of women dropped out and never met with skilled assistants at delivery (6). It is also lower than 46.5% dropout from institutional delivery and 87.1% from postnatal care visits in Ethiopia (9), as well as lower than 43.4% dropouts from ≥4 ANC to SBA and 85% from SBA to PNC in the maternal continuum of care (16). This might be attributable to older ages at the first birth, better awareness of healthcare services, a better level of education, and a favorable attitude toward maternal and child healthcare in the present study. In addition, it may be due to variation in the measurement of outcome variables. For instance, the aforementioned studies considered ANC dropout if women did not receive antenatal care at all, and SBA dropout if women gave birth out of a healthcare institution after antenatal care booking. On the other hand, the study found a higher prevalence, more than 32.2%, of dropout from the continuum of maternity care in Debre Markos town (17). This might be explained by the differences in the sociodemographic variables, study setting, sample size, variation in source data, and quality and accessibility of health facilities.

Women whose husbands were farmer was more likely to drop out of the continuum of care. Similar findings were reported in the Lao PDR (18) and northwest Ethiopia (8). Women whose husbands were farmer was less likely to complete the continuum of care. This might be because farmers have a low level of education, high workload, and lack of time to visit health institutions, as well as because most farmers live in rural areas, far from health facilities. The other reason might be that farmers have less access to information and media. In addition, in Ethiopia, men had more power and decision-making autonomy on healthcare than women.

Mothers in the low-income tertile had increased odds of continuum care dropout compared with those in higher income tertiles. In addition, these mothers were three times more likely to drop out of ANC visits. The poorest communities in Tanzania significantly dropped out of the care continuum and made fewer points of contact for care (7). Women from the poorest wealth quantile and residing in a community with high poverty were less likely to complete the recommended ANC contact (16). Although in Ethiopia, MNCH services are being provided for free, most women buy essential drugs on their own as these drugs are often not available in the facilities. The indirect costs for transportation, medication, and healthcare-related services might have contributed to the differences observed in the completion of CoC. Empowering women in terms of owning financial properties and safety net programs should be encouraged.

The number of children alive was found to be an important factor in the dropout from the maternity care continuum. Mothers who have two or three children alive were more likely to drop out of a continuum of care than those who have only one child. Secondary data analysis in Pakistan found that mothers having fewer children were most likely to avail of complete CoC (15). In Nepal, having more than two births reduced the odds of continuum care by 30% (19). Primiparous women might have high fear and/or perception of risk of complication and neonatal death. Primiparous women had high fear of childbirth and low childbirth self-efficacy than multiparous women (20).

The odds of dropout from a maternal continuum of care were four times higher among women who started the ANC follow-up later than those who started earlier. In addition, women who started the ANC follow-up after 16 weeks were less likely to receive full ANC and PNC visits. Women who started ANC visits within the first trimester were more likely to receive all MNCH services in the Lao PDR (18). In Gambia, initiating the first antenatal care within 16 weeks of pregnancy significantly determines the completion of maternity CoC (21). Early initiation of ANC increases the odds of completing the maternity care continuum in the North Shewa zone (10). Early ANC booking enhances positive pregnancy experience and increases access to adequate counseling on birth preparedness and complication readiness plan, place of delivery, birth companion, means of transportation, blood donor, and skilled birth attendants for the subsequent components of CoC, that is, skilled delivery and postnatal care. In addition, early ANC booking creates an active connection between the pregnant women and the healthcare provider; reduces healthcare service-related myths, fear, and stress; and increases the knowledge of danger signs during pregnancy, delivery, and the postpartum periods.

Women who spent approximately more than 15 min for ANC visits were two times more likely to drop out of the care continuum and PNC visits than those who spent <15 min. Longer time for consultation could affect satisfaction and completion of the maternity care continuum. In Peru, client satisfaction was higher among those who reported a consultation time of ≤ 15 min (22). Healthcare providers can enhance client satisfaction and utilization of all MNCH services by spending <15 min of discussion time with the client.

### Antenatal care dropout

Education increased the likelihood of higher intensity of continuum of care. Women whose partners attended a higher level of education were less likely to drop out of ANC visits. Education has also been shown to be associated with the completion of a continuum of care in previous studies (6, 8, 11, 21). More receptivity to new health-related information, better healthcare decision-making abilities, increased awareness of available health resources, ability to challenge negative norms/perceptions, good knowledge of danger signs, and the more financial resources of educated parents might have increased their likelihood of accessing and utilizing the maternal healthcare services. In most sub-Saharan African countries, including Ethiopia, men have high autonomy in the healthcare decision-making process and resource control and allocation. Hence, educated men are more likely to positively influence their women to complete the CoC.

Residency is an important predictor for the utilization of MNCH care services. The odds of antenatal care dropout were four times higher among rural residents. This is consistent with previous studies where urban residency is statistically significant with completion of maternity CoC (6, 21, 23). This is likely due to lack of timely transportation, long distance of health facilities, high workload, shortage of time, and costs involved. Even if health facilities are available in rural areas, there is a lack of healthcare providers, essential medication, and laboratory instruments (12). Furthermore, urban communities are more likely to be educated, and education was significantly associated with the continuum of care in our study.

Women who were not counseled about a continuum of care during ANC visits were more likely to have antenatal care dropouts. This finding was supported by previous studies (9, 11, 21), where antenatal counseling significantly decreased the dropout rate from ANC and skilled delivery. In addition, in Cambodia, utilization of four or more ANC visits was higher among women counseled by health providers (1). Counseling during ANC visits increases women awareness about the need for maternity care, danger signs of pregnancy, birth preparedness, and the possible hazards of inadequate care during pregnancy, which help develop willingness for the continuation of antenatal care services. In turn, this increases women's autonomy of healthcare decisions.

The odds of ANC dropout among women who were not satisfied with the service delivery increased 3-fold compared with those who were satisfied. In the North Gondar zone, the odds of completing the maternal healthcare services were higher among women who perceive satisfaction with the service delivery (11). In addition, perceived providers' poor reception of women was the main reason to have non-institutional deliveries in southern Ethiopia (24). This possible reason might be that satisfied women could have a positive attitude and health communications with healthcare providers, and this makes women more motivated to have full ANC contact, institutional delivery, and PNC contact.

### Postnatal care dropout

Mothers aged between 21 and 34 years had less risk of having PNC dropout than those older than 35 years. Similarly, mothers whose husbands were aged between 40 and 49 years were three times more likely to drop out of the PNC than those aged between 20 and 29 years. A National Health Extension Program assessment in Ethiopia showed that increasing age was negatively associated with the continuum of maternity care (25). Similarly, a community-based cross-sectional study in East Gojjam reported that being in the age range of 15–24 years increases the odds of early initiation and continuation of ANC visits (26). This could be possibly due to relative low childbearing experience and fear of complications and danger signs that may lead newly-wed adolescents to seek out PNC services. The current study has also found high PNC dropouts among grand multiparous women.

Women who were government employees were less likely to drop out of the PNC than those who were housewives. This was comparable with studies carried out in Arba Minch (27), northwest (8), and southern Ethiopia (24). The odds of completing the continuum of care were more likely among women whose husbands were employed. Employed husbands might have better information about the benefit of utilizing maternal health services, more financial resources, and a higher level of education, and hence, they might encourage their wives to use the service.

Mass media strongly predicts postnatal care utilization. Women who were not exposed to media were more likely to drop out of the PNC. The finding of a strong media effect is consistent with findings of previous studies (8, 9, 15–17). Mass media is an important technology that allows women to access maternal health-related information, the importance of regular health-seeking behavior, and challenge negative social norms, which helps women to improve their knowledge, attitude, and practice continuum of maternity care.

Grand multiparity significantly increased the odds of PNC dropout compared with primipara. Similarly, in Pakistan, having fewer children was significantly associated with the completion of maternity care (15). A secondary analysis of EDHS reported that increasing birth order increases the odds of dropout from institutional delivery (9). However, this finding is contrary to the study done in Northwest Ethiopia (8) and East Gojjam (26). The possible reason might be that nulliparous women might be sensitive to pregnancy-related complications and danger signs. The perceived risk of pregnancy on health tends to be lower among grand multiparous women. Thus, high parous women may lack the motivation to use maternal healthcare services, and are less likely to continue to use these services from pregnancy to postpartum period (19).

Moreover, in this study, the type of health facility was a significant determinant for dropout from PNC. Women who attend public health facilities had reduced odds of PNC dropout compared with those who attend private health facilities. There are no comparable studies for this finding. Public health facilities are affordable, and thus, women may prefer to initiate and continue maternity care at public health facilities. In addition, public health facilities might have better medical facilities than private institutions. Further research is needed to more closely examine the link between the type of health facility and PNC dropout.

## Conclusion and recommendation

Dropout from the continuum of care in the study area was high. Sociodemographic variables, media exposure, obstetrics, and health service-related factors were significant predictors of dropout from the continuum of maternity care. Socioeconomic development, partner involvement, antenatal counseling, efficient service delivery, and media exposure are vital to improving the high dropout rate from the maternal continuum of care. Furthermore, to reduce the burden of dropout from the care continuum, reproductive health officers and other health professionals should emphasize the importance of early first ANC visits.

## Limitation

The following are possible limitations that should be considered while interpreting the findings: First, the nature of the study design does not allow establishing temporal relationships, and second, social desirability and recall bias might have been introduced at the time of data collection.

## Data availability statement

The raw data supporting the conclusions of this article will be made available by the authors, without undue reservation.

## Author contributions

MT and SDT conceived and designed the study, performed analysis, and prepared the manuscript. DA, GEW, and GBM critically revised the manuscript, provided necessary comments, and made basic adjustments to the final manuscript. All authors gave final approval for publication.

## Conflict of interest

The authors declare that the research was conducted in the absence of any commercial or financial relationships that could be construed as a potential conflict of interest.

## Publisher's note

All claims expressed in this article are solely those of the authors and do not necessarily represent those of their affiliated organizations, or those of the publisher, the editors and the reviewers. Any product that may be evaluated in this article, or claim that may be made by its manufacturer, is not guaranteed or endorsed by the publisher.

## Abbreviations

ANC: antenatal care
AOR: adjusted odds ratio
CI: confidence interval
CoC: continuum of care
COR: crude odds ratio
EDHS: Ethiopia Demographic and Health Survey
ETB: Ethiopian birr
MNCH: maternal neonatal and child health
PNC: postnatal care
SBA: skilled birth attendant.
